# Cortical Complexity in People at Ultra-High-Risk for Psychosis Moderated by Childhood Trauma

**DOI:** 10.3389/fpsyt.2020.594466

**Published:** 2020-11-10

**Authors:** Jiaojiao Hou, Simon Schmitt, Tina Meller, Irina Falkenberg, Jianxing Chen, Jiayi Wang, Xudong Zhao, Jingyu Shi, Igor Nenadić

**Affiliations:** ^1^Shanghai East Hospital, Tongji University School of Medicine, Shanghai, China; ^2^Department of Psychiatry and Psychotherapy, Philipps-Universität Marburg and Marburg University Hospital, Marburg, Germany; ^3^Center for Mind, Brain, and Behavior, Philipps-Universität Marburg, Marburg, Germany; ^4^Tongji University School of Medicine, Shanghai, China; ^5^Shanghai Pudong New Area Mental Health Center, Tongji University School of Medicine, Shanghai, China; ^6^Division of Medical Humanities & Behavioral Sciences, Tongji University School of Medicine, Shanghai, China

**Keywords:** cortical complexity, magnetic resonance imaging (MRI), psychosis, schizophrenia, spherical harmonics, ultra-high risk (UHR)

## Abstract

Subjects with ultra-high risk (UHR) states for psychosis show brain structural volume changes similar to first-episode psychosis and also elevated incidence of environmental risk factors like childhood trauma. It is unclear, however, whether early neurodevelopmental trajectories are altered in UHR. We screened a total of 12,779 first-year Chinese students to enroll 36 UHR subjects (based on clinical interviews) and 59 non-UHR healthy controls for a case-control study of markers of early neurodevelopment. Subjects underwent 3T MRI scanning and clinical characterization, including the childhood trauma questionnaire (CTQ). We then used the CAT12 toolbox to analyse structural brain scans for cortical surface complexity, a spherical harmonics-based marker of early neurodevelopmental changes. While we did not find statistically significant differences between the groups, a trend level finding for reduced cortical complexity (CC) in UHR vs. non-UHR subjects emerged in the left superior temporal cortex (and adjacent insular and transverse temporal cortices), and this trend level association was significantly moderated by childhood trauma (CTQ score). Our findings indicate that UHR subjects tend to show abnormal cortical surface morphometry, in line with recent research; more importantly, however, this association seems to be considerably modulated by early environmental impacts. Hence, our results provide an indication of environmental or gene × environment interactions on early neurodevelopment leading up to elevated psychosis risk.

## Introduction

Psychotic disorders are often preceded by prodromal symptoms, leading to the construction of clinical criteria defining an “ultra-high risk” (UHR) status, which aims to facilitate early detection and intervention (1). UHR individuals might already present attenuated psychotic symptoms like brief subthreshold delusions and hallucinations (2). Additionally, deficits in social cognition and function have been found in at-risk states, which may contribute to the development of psychosis (3–5). Although nearly 30% of individuals at UHR transit into psychosis within the following 2 years, even non-transited UHR subjects might still suffer from continued attenuated psychotic-like symptoms and social functional impairment (6). Thus, identifying potential risk factors and illuminating the biological mechanisms are essential for the development of early target intervention.

Previous studies have demonstrated that UHR status or risk is associated with environmental exposures such as urbanicity (7), negative life events (8), cannabis use (9), and childhood trauma (10). In particular, maltreatment during childhood is strongly related to heightened social stress sensitivity (11), clinician-rated positive (but not negative) symptoms in at-risk youth (12) and occurrence and persistence of psychotic symptoms across the psychosis spectrum (13). These findings suggest that childhood trauma might be an essential contributor to the development of psychosis. However, its neurobiological mechanisms are still unknown.

Both UHR status or risk for psychosis and early environmental stressors like childhood trauma show effects on brain structure, thus mediating genetic and non-genetic impacts on this putative biomarker. Multiple studies have shown the overlap (and differences) of brain structural effects in case-control studies in UHR and first-episode psychosis (14), including prefrontal as well as medial temporal brain volumes. Indeed, early childhood trauma might affect brain volumes/structure in areas affected by psychosis, such as the hippocampus or corpus callosum (15). In schizophrenia patients, childhood trauma has been associated with reduced dorsolateral prefrontal cortex (DLPFC) volume (16), as well as reduced amygdala (17) and hippocampus volumes (18). [For review see (19)] However, regional volume changes assessed through volumetry or voxel-based morphometry (VBM) are likely to represent cumulative effects of genetic, environmental, and other effects. It thus remains unclear, whether brain structural effects of childhood trauma in individuals with UHR states reflect the sequelae of subtle neurodevelopmental abnormalities as seen in schizophrenia.

Surface-based morphometric markers have been used to tap more specifically into the early developmental aspects of brain morphometry. These studies have shown subtle cortical folding abnormalities in neocortical areas overlapping with those seen in VBM studies of schizophrenia (20–22). More recently, abnormalities of cortical surface features have also been shown in clinical high-risk youth with a prodromal syndrome (23). However, it remains unclear whether early environmental stressors like childhood trauma might interact with other (e.g., genetic) factors resulting in abnormal surface morphology.

In this study, we propose the use of cortical complexity (CC), a surface-based morphometry (SBM) method (24, 25) to measure the brain structural changes in ultra-high risk states for psychosis. While some previous studies in UHR focused on help-seeking populations, we chose a community-driven approach screening a large number of students (*n* = 12,779), and subsequently identifying UHR subjects based on clinical interviews. In particular, the aim of this study was to (1) use cortical complexity to estimate the shape of cortical surfaces in high risk psychosis in college students, and (2) explore the possible interaction and underlying mechanisms between childhood maltreatment and altered folding in high risk for psychosis, in order to evaluate the impact of a prominent environmental risk factor and predictor of subsequent psychopathology.

## Methods

### Study Cohort and Recruitment Procedure

Based on screening *n* = 12,779 Chinese students from Tongji University in Shanghai, China, we recruited a sample of 95 subjects for MRI analyses, including 36 UHR subjects and 59 non-UHR healthy controls. Subject recruitment and inclusion into the study followed a protocol approved by the ethics committee of the Institutional Review Board at Tongji University.

Subject recruitment was based on the psychometric screening of large cohorts of undergraduate (1st-year) Tongji University students, who first underwent screening using the prodromal questionnaire (PQ-16 or PQ-B), identifying potential UHR subjects, who then received a clinical interview [Semi-structured Interview for Psychosis risk Syndromes (SIPS)], and upon confirmation were invited to participate as UHR subjects, while otherwise healthy non-UHR subjects from the same population were randomly recruited from that screening cohort.

Recruitment was conducted in three waves/study cohorts:

In the first wave, the PQ-16 was distributed to 4,040 undergraduate students (with 3,121, 77.3% questionnaires returned). The cut-off score of PQ-16 was set at 9, and 71 participants reached the cut-off score (2.3% of the screening population). We invited these participants to our second stage assessment including the SIPS interview, which was conducted by trained psychiatrists. Twenty-nine participants (40.8%) met the criteria as being at ultra-high risk for psychosis based on the SIPS results. One year later, these participants were re-assessed using SIPS (for inclusion in the MRI study). Ten participants still met the UHR criteria while one participant was diagnosed with bipolar disorder. These 10 UHR completed the study including MRI scanning.

In a second wave, the PQ-B was given to 4,364 undergraduate students (3,498, 80.2% questionnaires returned). Applying the PQ-B with a cut-off score of 24, according to a previous study (26, 27), 1,364 participants (39.0%) reached this threshold, of which 16 participants met UHR criteria following a SIPS interview (1.2%). One participant refused MRI scanning, another 15 individuals meeting UHR criteria completed the study including MRI scanning in this population.

In a third wave, the PQ-16 was distributed to 4,675 undergraduate students [3,903 (80.2%) questionnaires returned], of which 100 participants (2.6%) reached the cut-off score of 9. After the second stage assessment using SIPS, 20 participants met the UHR criteria (20%). One participant dropped out and eight participants refused to participate in MRI scanning. Hence, eleven participants of this population completed the study including MRI scans.

All SIPS interviews were conducted within 1 month after finishing first stage assessment. Participants completed MRI scans within two months.

Healthy controls were selected randomly out of those with scores below cut-off at first stage assessment at Tongji University.

General exclusion criteria included previous psychiatric diseases or treatment, drug-abuse, psychotropic medication and family history of schizophrenia.

This community-based two-stage recruitment procedure (and exclusion of subjects unsuitable for morphometric analysis, see below) resulted in a study sample of a total of 95 subjects: 36 UHR subjects (24 men, 12 women; mean age = 19.08, SD = 0.73) and 59 healthy non-UHR subjects (32 men, 27 women; mean age = 20.49, SD = 1.12). All subjects were Chinese.

### Questionnaires for Initial UHR Screening

As outlined above, the large-scale recruitment used the prodromal questionnaire (PQ) as a screening tool to identify at-risk subjects from the student community who were subsequently invited for clinical interviews to confirm UHR criteria. Due to a change in the protocol of this large on-going Tongji high-risk study, two slightly different versions of the PQ were used in the different waves of recruitment of undergraduate classes.

The 16 items prodromal Questionnaire (PQ-16) is a screening tool for prodromal symptoms of psychosis (27). It consists of 9 items from the hallucinations and perceptual abnormalities subscales, 5 items concerning paranoia, delusional ideas and unusual thought content and 2 negative symptoms. Although the cut-off score of 6 or more has been considered “positive” in previous studies (27), we found the cut-off score of 9 or more has higher sensitivity (68%) and specificity (73%) in Chinese college students (28).

The 21-item prodromal questionnaire-brief version (PQ-B) is a self-report assessment to screen the psychosis at-risk state, comprised of positive symptom items plus follow-up questions about related distress/impairment on a scale from 1 to 5. A total score of 24 or more will be considered “positive.” The Chinese version of PQ-B was validated and has high sensitivity (83.8%) and specificity (60.9%) (29).

### Clinical Assessment of UHR Status Using SIPS Interviews

The semi-structured interview for psychosis risk syndromes (SIPS) was administered in this study by trained psychiatrists to ass ess risk of psychosis/UHR status (30). The SIPS consists of the Scale of Prodromal Symptoms (SOPS) and the incorporated Criteria of Prodromal Syndromes (COPS). The severity of symptoms is assessed by SOPS on four main scales: positive, negative, disorganized and general symptoms. An “attenuated psychotic symptom” is identified when at least one positive symptom is rated over 2 but <6 (ranging from 0 to 6). The Chinese version of SIPS has been shown to provide good reliability and validity (31).

### Assessment of Childhood Trauma

Subjects completed the short form of Childhood Trauma Questionnaire (CTQ-SF), a 28-item self-reported questionnaire measuring the experience of childhood trauma. The scale considers five subscales reflecting basic aspects of childhood traumatic experiences: emotional neglect, physical neglect, emotional abuse, physical abuse, and sexual abuse. Each subscale includes five items and the scores of each item are obtained on a 5-point Likert scale (1–5, i.e., “never” to “very often”). The Chinese version of CTQ-SF shows good cultural equivalence, good reliability and validity (32).

### Magnetic Resonance Imaging (MRI) Data Acquisition

Structural images were acquired with a 3.0 T MR scanner (General Electrics GE MR750) located at Tongji University, Shanghai, China. Structural data were collected using a 3D FSPGR (fast spoiled gradient-echo) sequence (voxel size: 1 × 1 × 1 mm^3^, field of view: 192 × 192 mm, 136 slices, TR = 8.168 ms, TE = 3.172 ms, TI = 450 ms, α = 12°).

### MRI Data Preprocessing

MRI data were preprocessed with CAT12 (version 1184; Christian Gaser, Jena University Hospital, Germany; http://www.neuro.uni-jena.de/cat/), a toolbox for SPM (33) that includes a pipeline for surface based morphometry. Cortical surfaces were extracted from the T1 scans by using a spherical harmonics approach (34). Topological corrections were applied (35) and surfaces were spherically mapped with a volume-based diffeomorphic DARTEL algorithm (36). Then, using a fractal dimensions approach (35) local surface complexity was estimated for each participant. These datasets were smoothed with a Gaussian kernel of 20 mm full width at half maximum (FWHM).

In order to check for data quality homogeneity checks were conducted with the CAT12 toolbox, and since three data sets did not pass the imaging assurance protocol (due to low intercorrelations arise from head movements), these had been excluded from further analysis.

### Statistics

Using the general linear model (GLM) framework of SPM, our first statistical analysis was a group comparison of UHR vs. non-UHR subjects to test for differences in cortical complexity on a vertex-level across the entire brain, equalling *T*-tests for both directions (i.e., cortical complexity increases and decreases in UHR relative to non-UHR). We first corrected for multiple (vertex-level) comparisons using a threshold of *p* < 0.05 with peak-level family wise error correction (FWE) (37); We then performed the Threshold-Free Cluster Enhancement (TFCE) option based on 5,000 random permutations; after that we repeated the tests at *p* < 0.001 uncorrected thresholds, since for the testing of our second hypothesis (i.e., a moderation of the association of UHR status and regional cortical complexity through childhood trauma), we also needed to consider the possibility of regional effects, where the direct association of UHR status and regional CC might not reach statistical significance (on the vertex-level), which might be explicitly explained by a moderation model. Hence, while this uncorrected threshold does not correct for the direct association of UHR status and CC, it considers the possibility of regional associations that are moderated by a third variable (i.e., CTQ-SF) and thus insignificant in direct association. For automated anatomical assignment of identified clusters, we used the Desikan-Killiany-40 atlas (25, 38–40).

Moderation analysis for testing our second hypothesis was then performed on the basis of clusters identified in the first (vertex-level) analysis. To test the assumption that the level of childhood maltreatment modulates the association of UHR-status and cortical complexity, we set up a moderated logistic regression model using the PROCESS macro v3.4 running under SPSS (IBM Statistical Package for Social Sciences, version 24, IBM, Armonk, NY). For that, we extracted eigenvariate values from the significant cluster detected in the SBM analysis and entered that variable as predictor, UHR-status as outcome variable and CTQ sum score as moderator. In the model, age and sex were included as covariates. The moderating effect was tested both for the CTQ sum score and subsequently, for each of its sub scores.

## Results

### Demographic Data

We found that the UHR/non-UHR groups did not differ regarding gender ratio M/F (Chi-square, *Chi*^2^ = 1.427, *p* = 0.232), the ratio of handedness R/L (*Chi*^2^ = 3.140, *p* = 0.076) and the ratio of ethics Han/other ethnic groups (*Chi*^2^ = 0.033, *p* = 0.855). UHR individuals were younger than healthy controls (ANOVA *F* = 45.065, *p* < 0.001, *d* = 1.259). As we recruited participants in the same college, we do not expect too many differences concerning education background between the two groups. For the UHR group, the scores of four subscales for SIPS including positive symptoms (mean value = 8.20, SD = 3.71), negative symptoms (mean value = 4.77, SD = 5.02), disorganized symptoms (mean value = 2.80, SD = 2.93) and general symptoms (mean value = 3.86, SD = 3.33). The demographic information and scores of SIPS are presented in Table 1.

**Table 1:** Demographic and Clinical Characteristics.

	**UHR** **(*n* = 36)**		**non-UHR HC** **(*n* = 59)**		**Statistics**	
	**Mean**	**SD**	**Mean**	**SD**	***F*/*x*^**2**^**	***p***
Age (year)	19.08	0.73	20.49	1.12	45.065	<0.001
Gender			32/27		1.427	0.232
Male	12 (33.3%)		32 (54.2%)			
Female	24 (66.7%)		27 (45.8%)			
Handedness					3.14	0.076
right	22 (61.1%)		25 (42.4%)			
left	14 (38.9%)		34 (57.6%)			
Ethnicity	31/5		50/9		0.033	0.855
Han	31 (86.1%)		50 (84.7%)			
Other	5 (13.9%)		9 (15.3%)			
SIPS	Mean	SD				
Positive	8.2	3.71				
Negative	4.77	5.02				
Disorganized	2.8	2.93				
General	3.86	3.33				
**CTQ**						
Emotional abuse	8.56	3.17	7.27	3.08	0.216	0.054
Physical abuse	6.44	3.85	5.90	1.53	6.273	0.333
Emotional neglect	11.94	4.71	9.41	4.24	0.728	0.008
Physical neglect	8.06	3.26	6.83	2.65	3.520	0.048
Sexual abuse	5.06	0.33	5.49	1.10	22.788	0.024
Total	40.06	10.57	34.90	9.44	1.625	0.015

*UHR, Clinical ultra-high risk state for psychosis; HC, healthy control; SIPS, the Structured Interview for Prodromal Syndromes*.

### Cortical Complexity Group Differences UHR vs. non-UHR

**Figure 1:**
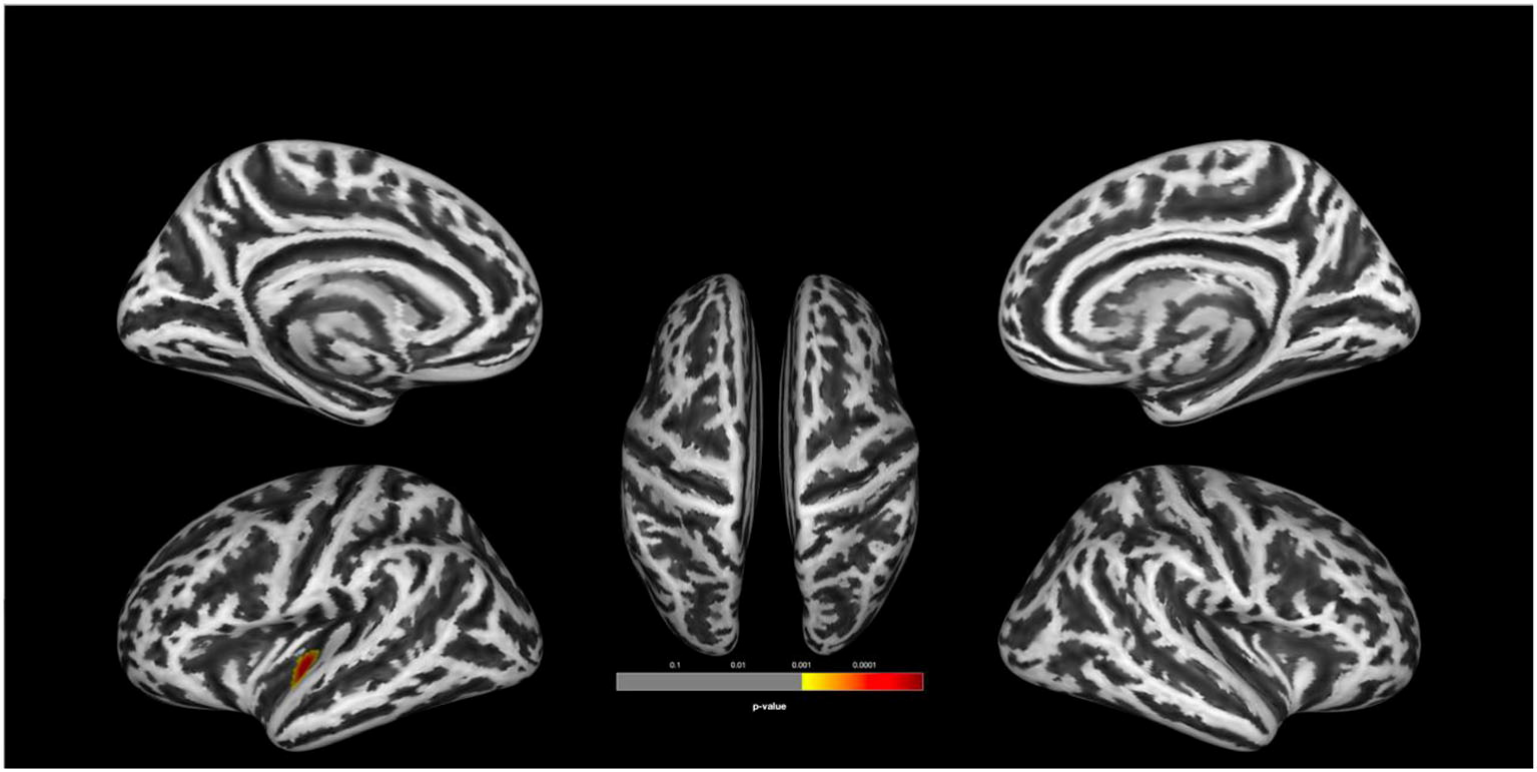
Group comparison indicating lower cortical complexity in ultra-high risk for psychosis (UHR) vs. non-UHR healthy controls (HC). The clusters are thresholded at *p* < 0.001 (uncorrected). Display of trend level findings: While there were no statistically significant (*p* < 0.05, FWE cluster level corrected) group differences, we observed a trend level finding for reduced cortical complexity in a cluster in the left superior temporal gyrus, extending toward the insular and transverse temporal cortices. The above image is displayed at a *p* < 0.001 uncorrected threshold for display purposes.

There was no statistically significant (*p* < 0.05, corrected for multiple comparisons, FWE cluster level corrected) between the two groups. TFCE analysis also did not show statistically significant group level effects for cortical complexity. However, we observed a trend level finding at *p* < 0.001 uncorrected levels in a cluster comprising the left superior temporal gyrus (STG) and parts of the left insular and transverse temporal cortex. In addition, uncorrected analyses also identified two smaller clusters of relative increases of CC in UHR vs. non-UHR in the left supramarginal and left rostral middle frontal cortices. Findings are shown in Table 2 and Figure 1.

**Table 2:** Cortical complexity findings (*p* < 0.001 uncorrected levels) comparing ultra-high risk (UHR) for psychosis subjects vs. non-UHR healthy controls (HC); k: numbers of vertices in cluster.

**Contrast**	**MNI**	**Atlas labeling DK 40**	***k***	***P*-value**	***z*-Score**
UHR < HC	−44; −20; −6	Left superior temporal (82%), insula (15%), transverse temporal (3%)	101	3.295 × 10^−5^	3.99
UHR>HC	−35; −38; 38	Left supramarginal	9	8.167 × 10^−4^	3.15
	−27; 34; 31	Left rostral middle frontal	6	8.704 × 10^−4^	3.13

### Moderation Analysis With Childhood Trauma

Based on the results of the analysis of cortical complexity, we tested whether the association of CC variation and UHR vs. HC status found for a cluster within the superior temporal gyrus would be moderated by the level of childhood maltreatment reported by the participants. We first tested this assumption in a model including CTQ sum score as moderating variable. This model is overall significantly better than a constant-only model [χ^2^(5) = 64.19, *R*^2^(McFadden) = 0.509, *p* < 0.001] as are both the main effects of CC values (*b* = −12.890, *p* = 0.001) and CTQ sum (*b* = 0.093, *p* = 0.015) and the interaction of the two (*b* = 0.636, *p* = 0.039). Further, including the interaction improves the model significantly [χ^2^(5) = 5.024, *p* = 0.025]. The positive coefficient of the interaction term indicates the effect of CC becoming more positive (i.e., decreasing) with increasing CTQ sum score.

**Figure 2:**
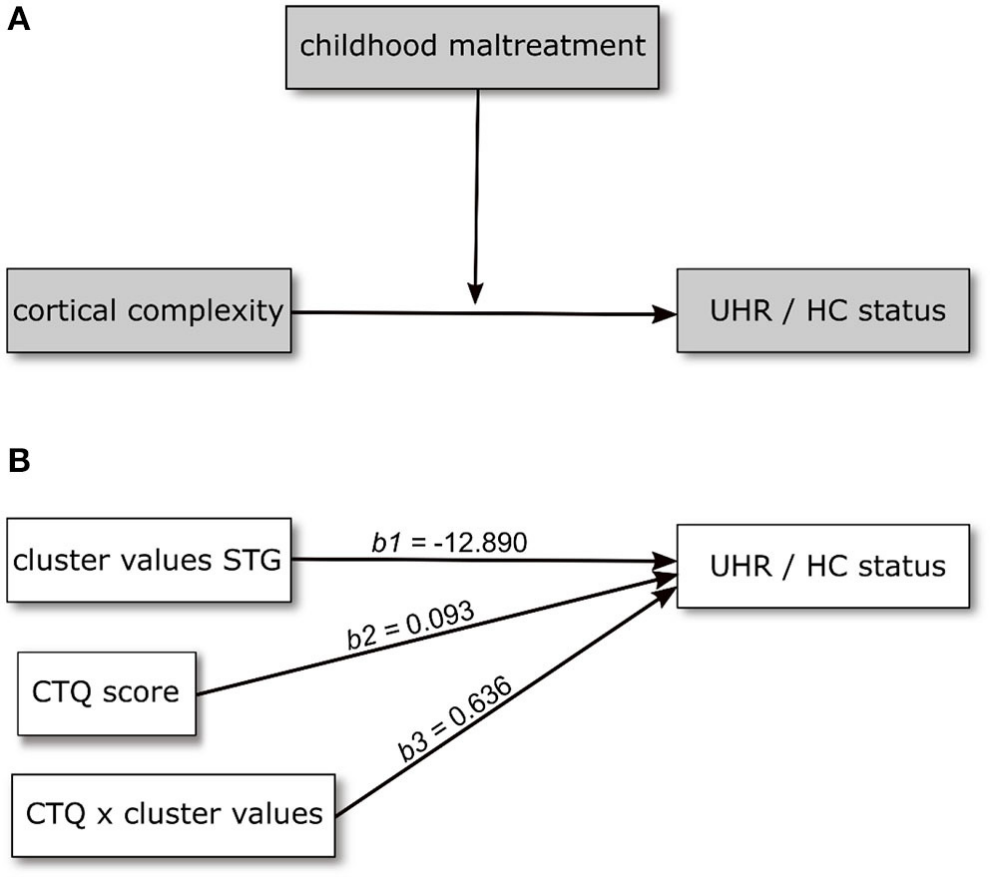
Conceptual **(A)** and statistical **(B)** model showing the relationship between cortical complexity and UHR/HC status being moderated by level of childhood maltreatment. Regression coefficients in **(B)** are calculated in a model including age and sex as covariates.

**Figure 3:**
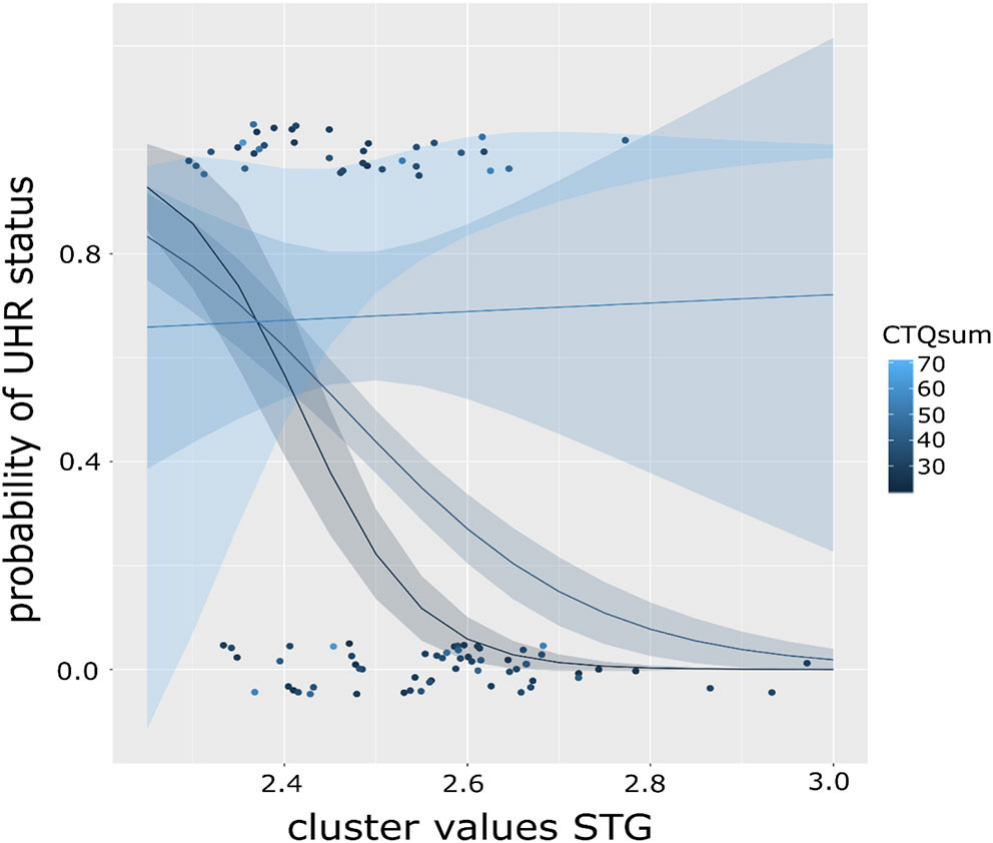
Graph showing the moderation effect of level of childhood maltreatment (CTQ) on the association of cortical complexity in the superior temporal gyrus (STG) and the probability of UHR status in a binominal logistic regression model. Color of the dots indicates CTQ scores, lines represent fitted regression models dependent on CTQ sum score, exemplary for CTQ score of 20 (black), 40 (dark blue), and 60 (light blue) with respective standard error intervals. The steepest curve (i.e., strongest association) between STG CC and UHR status is present at low CTQ scores and decreasing with increasing CTQ scores (as shown in the flattening curve).

We then explored the effect on the level of CTQ dimensions to determine if they contribute differently to the moderating effect. Here, only the model including the emotional neglect (EN) scale is significant [χ(5) = 71.64, *R*^2^ (McFadden) = 0.568, *p* < 0.001], as is the main effect of EN (*b* = 0.352, *p* = 0.003) and its interaction with CC (*b* = 2.496, *p* = 0.009). This model, too, is improved by inclusion of the interaction [χ^2^(5) = 9.697, *p* = 0.002]. It should be noted that multiple testing correction were not performed. Results were presented in Figures 2, 3.

## Discussion

This study provides first evidence of a moderating effect of childhood trauma on the emergence of subtle cortical folding abnormalities related to the UHR state for psychosis. In the search of viable biomarkers of risk for psychosis, this provides an important step as it calls for a more detailed consideration of environmental risk factors impacting on early brain development assumed to largely be driven by genetic factors. Three main aspects of this study deserve particular attention in our discussion: first, the identification of subtle cortical folding alterations distinguishing clinically healthy UHR subjects from their non-UHR controls, second, the moderation of these effects by childhood trauma, which implies that different levels of trauma modulate the relationship between UHR status and cortical complexity, and third, a decreasing score of emotional neglect strengthened the effects of cortical complexity in STG, a key area related to risk for schizophrenia.

Cortical complexity is an SBM method that could reflect the pathogenic processes in cortical structure changes, and has been used successfully to measure the aberrant cortical folding in schizophrenia (25, 39, 40) and bipolar disorder (41). The main development of cortical folding seems to happen during the fetal period and the basic folding pattern remains stable across the lifespan (42). This indicates that CC is more related to pre- and postnatal factors like intra-uterine infections, obstetric complications and genes (43–45). In other words, changes in cortical complexity may be related to an individual's genetic vulnerability to develop psychosis (46, 47). Our findings expand on this pathophysiological understanding by showing the additional impact of early childhood environmental risk.

We also found that ultra-high risk individuals showed reduced cortical complexity in the superior temporal gyrus (STG) and insula in the left hemisphere, corresponding with previous studies that found differences of gyrification in UHR (23). STG and insula are essential for auditory perception, cognitive functions, thought and language processing. Previous studies have found that the morphological changes of STG and insula in UHR may be related to the premorbid language abnormalities (48–50). Longitudinal imaging studies also found that STG in UHR individuals who later transitioned to and higher risk of transition into psychosis psychosis showed a greater decrease in cortical gray matter (51), which indicates that morphometric abnormalities of STG in UHR may be an essential region for the underlying mechanisms of psychosis onset (52, 53). Additionally, as cortical complexity reflects the early deficits rather than the development of the diseases state, our results indicate that STG and insula may play an essential role in the genetic vulnerability of onset psychosis. Previous studies on genetic risk for psychosis also found that abnormalities of cortical gyrification in STG were negatively correlated with positive prodromal symptoms (54) and persistence of psychotic experience (55). Therefore, these morphological changes in STG seem to be related to the clinical symptoms, especially positive psychotic symptoms in high risk for psychosis, and STG may be one of the essential regions for the underlying neuromechanisms of genetic vulnerability to develop psychosis.

Moderation analysis has been used to address that whether and to what degree the effects of one variable on an outcome exists under different levels of an additional condition (56). The moderating effect of childhood maltreatment was observed between variation in cortical complexity and UHR/HC status. Our finding of a significant main effect of childhood trauma further establishes its role as an environmental risk factor for UHR in college students, which is supported by previous studies (10, 57, 58).

Interestingly, our moderation analysis suggests that the effect of cortical complexity on UHR *decreases* with higher childhood trauma. In other words, genetic factors may make individuals more vulnerable to the influences of environmental factors, but this may also depend on the degree of adverse exposures (59). Several studies hypothesize that the onset of psychosis may be triggered by both the influences of genes and environmental risk factors (60, 61), but the mechanisms of gene-environment interaction are still unclear. Our study addressed the effects of environmental contribution to psychosis, especially when the degree of trauma exposure was high. Previous hypotheses postulate that environmental factors and genes may impact on a final common pathway, like sensitizing the dopamine system, causing progressive dysregulation and developing into psychosis (62). Additionally, we found that the effect of cortical complexity in STG decreased under high levels of emotional neglect, which suggests that emotional neglect may play a more important role in moderating the effect of cortical complexity on UHR status compared to other subscales in childhood trauma. In sum, our finding provides more evidence to elucidate the combining influences of genes (as cortical folding is significantly regulated by multiple developmental genes) and environmental factors for the onset of psychosis (63).

On a phenotype level, the interaction of genetic liability and childhood trauma has been shown for the emergence of psychotic symptoms (64), as well as UHR (65–67). Previous research has implied changes in gyrification (in non-psychotic subjects) to be related to childhood abuse experience (68). Our study links these lines of evidence showing that childhood trauma has a moderating effect on the association of UHR status with cortical complexity; this suggests a potential clue for the interaction of genetic effects (controlling brain development) and environmental stress. Given that types of childhood maltreatment also alter the structural connectivity along major white matter tracts (69), it is conceivable that this might also affect the later stages of cortical folding in the child brain. However, further research would be necessary to link molecular genetic markers of brain development and psychosis risk in their interaction with childhood maltreatment. In particular, the timing of both late (post-natal) cortical folding and vulnerable periods for the impact of environmental stressors are not well understood.

The lack of more wide-spread cortical folding changes in UHR subjects, esp. in regions that might pre-dispose to later volume loss in transition to psychosis, might be attributable to a number of factors. As suggested by our study, the direct effect might be moderated by a third variable, of which our study only tested childhood maltreatment. It is conceivable that other environmental stressors not assessed in this analysis might also modulate the relation between cortical folding and UHR status, prompting inclusion of additional environmental parameters for future studies. A second factor to consider is that cortical folding abnormalities might not necessarily result in subsequent volume loss upon disease onset: first-episode psychosis is likely to affect multiple neural networks and preceding neurodevelopmental effects might be a predisposing, but not necessary prerequisite for atrophy-like progression.

We also need to consider some methodological limitations, however. Despite screening a very large cohort of students, our UHR sample was rather limited. While typically with a sample of 50 vs. 50 subjects one would expect to detect effect sizes of *d* = 0.5 (assuming power 0.8 and alpha 0.05), our sample sizes would be expected to achieve 0.75 power (with alpha 0.05 and D = 0.53 effect size; as calculated by GPower3.1 sensitivity analysis). This would imply that at least medium to larger effects should have been detected with our sample at reasonable power assumptions. However, limited sample size might have precluded detection of more subtle effects. This limitation has to be outweighed against the unique characteristics of the sample: UHR subjects were recruited from a non-help seeking population (hence limiting the bias introduced by subjective distress related to UHR-symptoms) and are more homogeneous for sociodemographic factors (as they stem for a pre-defined student population). Another major limitation of our interpretation linking fugitive genetic effects to our findings is that even though cortical complexity might be related substantially the effects of genes implicated in early brain development, additional environmental factors can impact on the formation of cortical folds, including our findings. Also, the choice of cortical complexity would be expected to focus on the part of brain structural variance related to early development aspects (as opposed to brain volumes, which are influenced by a larger number of factors). Yet, even though cortical complexity might be a more sensitive parameter, further replication of our findings is warranted. Since our study only involved cross-sectional data, we cannot infer on the relation of either cortical folding itself or the moderation effect in related to transition rates.

In conclusion, this study detected differences of cortical surface folding between individuals at ultra-high risk for psychosis and healthy controls using cortical complexity. Childhood trauma, however, is a significant modulator in this association, suggesting that early brain development leading up to UHR is significantly influenced by (additional) environmental stressors, even for markers of aberrant early neurodevelopment. However, a robust significant association between cortical folding and later UHR status might depend on additional environmental risk factors and thus might be absent in subjects without the latter.

## Data Availability Statement

The original contributions presented in the study are included in the article/supplementary materials, further inquiries can be directed to the corresponding author/s.

## Ethics Statement

The studies involving human participants were reviewed and approved by the Institutional Review Board at Tongji University. The patients/participants provided their written informed consent to participate in this study.

## Author Contributions

JH: data acquisition, data analysis, original draft preparation, and manuscript writing. SS and TM: data analysis and manuscript writing. IF: manuscript revision and editing. JC: data acquisition and manuscript editing. JW: data analysis and manuscript revision. XZ: manuscript editing. JS: funding acquisition. IN: supervision of data analysis, data interpretation, original draft preparation, manuscript writing, and revision. All authors contributed to the article and approved the submitted version.

## Conflict of Interest

The authors declare that the research was conducted in the absence of any commercial or financial relationships that could be construed as a potential conflict of interest.
